# A non-fluorinated superhydrophobic composite coating with excellent anticorrosion and wear-resistant performance

**DOI:** 10.3389/fchem.2022.952919

**Published:** 2022-10-03

**Authors:** Peng Xiao, Liheng Yang, Jianjun Liu, Xiaoqin Zhang, Dabing Chen

**Affiliations:** State Grid Jiangsu Electric Power Co., Ltd. Research Institute, Nanjing, Jiangsu, China

**Keywords:** fluorine-free, graphene, superhydrophobic coating, wear resistant, anticorrosion

## Abstract

The facile and low-cost fabrication of fluorine-free superhydrophobic metal surfaces for anticorrosion remains a challenging issue. Here, we report a superhydrophobic coating based on polyacrylate/SiO_2_ nanoparticles/graphene oxide sheets through a simple yet environmentally friendly method. The as-prepared composite coating sprayed on metal surfaces exhibits excellent superhydrophobic and corrosion-resistant properties. Furthermore, the coating surface possesses good anti-wear performance and remains superhydrophobic after harsh abrasion tests. Prospectively, the developed non-fluorinated superhydrophobic coating opens up opportunities for the application in industrial anticorrosion field.

## Introduction

Among various functional coatings, the superhydrophobic surface with a water contact angle greater than 150° and a sliding angle less than 10° is growing in popularity (Fihri et al., 2017; Ghasemlou et al., 2019; Nguyen-Tri et al., 2019; Hooda et al., 2020; Wang and Urban, 2020). The artificial superhydrophobic surfaces offer exciting prospects related to self-cleaning (Latthe et al., 2019a; Dalawai et al., 2020), oil–water separation (Wang et al., 2019; Kang et al., 2021), anti-icing (Latthe et al., 2019b), drag reduction (Xu et al., 2020), corrosion inhibition (Shi et al., 2019; Wei et al., 2021), and anti-biofouling (Sun et al., 2018) properties. Generally, superhydrophobic surfaces possess high roughness and low surface energy, and several methods have been reported to realize superhydrophobization (Bayer, 2020).

Previously, the superhydrophobic coatings were usually prepared based on fluoropolymers such as perfluorooctanoic acid and perfluorooctyltrichlorosilane. This could be attributed to the perfluoroalkyl surface treatments (also termed fluorination) that can substantially reduce the surface free energy of the coating. Nevertheless, recent research has demonstrated that long-chain polyfluorinated compounds could lead to bioaccumulation and potential harm to human offspring (Ferrari et al., 2019; Ferrari et al., 2021). Therefore, non-fluorinated materials with the advantages of nontoxicity and low cost are preferable for the design and fabrication of superhydrophobic coatings. Graphene, a single-atom-thick layer composed of *sp* ^2^-hybridized carbon atoms, exhibits many distinctive merits in electronics, chemistry, and physics. Due to their chemical inertness and huge specific surface, graphene materials also have revealed the potential in novel anticorrosion coatings in recent years (Naderizadeh et al., 2018; Mao et al., 2020).

In this study, we develop a non-fluorination superhydrophobic coating by combining acrylate copolymer, SiO_2_ nanoparticles (SiO_2_ NPs), and graphene oxide (GO) sheets. Particularly, the polyacrylates containing ester-bonded copolymers enable low surface energy and wear resistance. The GO sheets dispersed in the coating can act as a barrier against corrosive media. The modified SiO_2_ NPs cooperated with GO forming micro-/nanostructures on the coating surface to produce high roughness, further improving the hardness and wear resistance of the coating. The obtained composite coating possesses remarkable superhydrophobicity and exhibits excellent wear-resistant and anticorrosion performance on the metal surface. Given the superior performance, as well as facile and environmentally friendly preparation, the superhydrophobic coating offers a unique opportunity for practical anticorrosion applications in the future.

## Experimental

### Materials and methods

Tetraethylorthosilicate (Si(OCH_2_CH_3_)_4_, TEOS), ethanol, methyl methacrylate (MMA), n-butyl acrylate (BA), acrylic acid (AA), azobisisobutyronitrile (AIBN), n-butanol, and cetyltrimethoxysilane were all provided by HEOWNS Chemical Reagent Co., Ltd. (Tianjin, China) and used without further processing (purity of ≥98.0%). GO sheets were purchased from Chengdu Organic Chemicals Co., Ltd (purity of 99%).

### Preparation and modification of SiO_2_ NPs

The modified superhydrophobic SiO_2_ NPs were prepared through a one-pot sol-gel process. In a typical procedure, 33 ml of ethanol and 18 ml of ammonium water were evenly mixed at 64°C. After adding 34 ml of TEOS, the mixture was then placed in an oil bath and stirred for 6 h. Next, 2 ml of cetyltrimethoxysilane was added to the mixture, and the total reaction system was stirred for another 2.5 h. After that, the mixture was vacuum filtrated with a Buchner funnel. The residues were rinsed with ethanol and deionized water and then dried at 120°C.

### Synthesis of acrylate copolymer/SiO_2_–NPs/GO sheet composites

The acrylate copolymer was synthesized *via* radical polymerization. First, 50 ml of tetrahydrofuran was added in a 500 ml three-necked round-bottom flask under a nitrogen flow for 10 min to remove oxygen. Next, the flask was placed in an oil bath and with mechanical stirring using a Teflon bar. Monomers of 25 g MMA, 20 g BA, 15 g AA, and 0.2 g AIBN were added dropwise into the solution. The mixture was stirred at 70°C for 8 h under constant nitrogen flow to complete the polymerization reaction. Afterward, the product was recrystallized three times with excess n-hexane and then prepared with absolute ethanol to a concentration of 0.1 g/ml. A certain amount of SiO_2_ NPs and GO sheets were sequentially added into the solution, followed by a low-power sonication and the acrylate copolymer/SiO_2_–NPs/GO sheet composite coating was obtained.

### Characterizations

The water contact angles (WCAs) and rolling contact angles (WSAs) of the surfaces were measured using an optical contact angle meter (Data physics, OCA 20) with a drop volume of 5 μl at ambient temperature. The morphologies of the composite coatings were observed by scanning electron microscopy (SEM, FEI Nova Nano SEM450) and using a 3D profiler. The particle size of the synthesized SiO_2_ NPs was determined using the Particle Sizer and Zeta Potential Analyzer (90Plus PALS, Brookhaven). Thermogravimetric analysis (TGA) was carried out on a NETZSCH thermal gravimetric analyzer (TG209F3). X-ray photoelectron spectroscopy (XPS) was tested by ESCALAB 250 (Thermo Fisher Scientific, United States). Durability evaluation of the superhydrophobic coatings sprayed on a Tinplate (50 mm × 50 mm) was performed using 800 mesh sandpaper with a 200 g loading. The anticorrosion performance was examined using an electrochemical workstation (CHI 760E, CH Instruments, Inc.). A three-electrode cell with a saturated calomel electrode (SCE) as the reference, a platinum electrode as the counter, and the samples with an exposed area of 1 cm^2^ as the working electrode were immersed in a 3.5 wt% NaCl solution for 30 min to stabilize. The potentiodynamic polarization curves were measured at a sweep rate of 10 mV/s, and electrochemical impedance spectroscopy (EIS) curves were measured with a sinusoidal perturbation signal of 5 mV amplitude and a frequency ranging from 10^−2^ Hz–10^5^ Hz.

## Results and discussion

**FIGURE 1:**
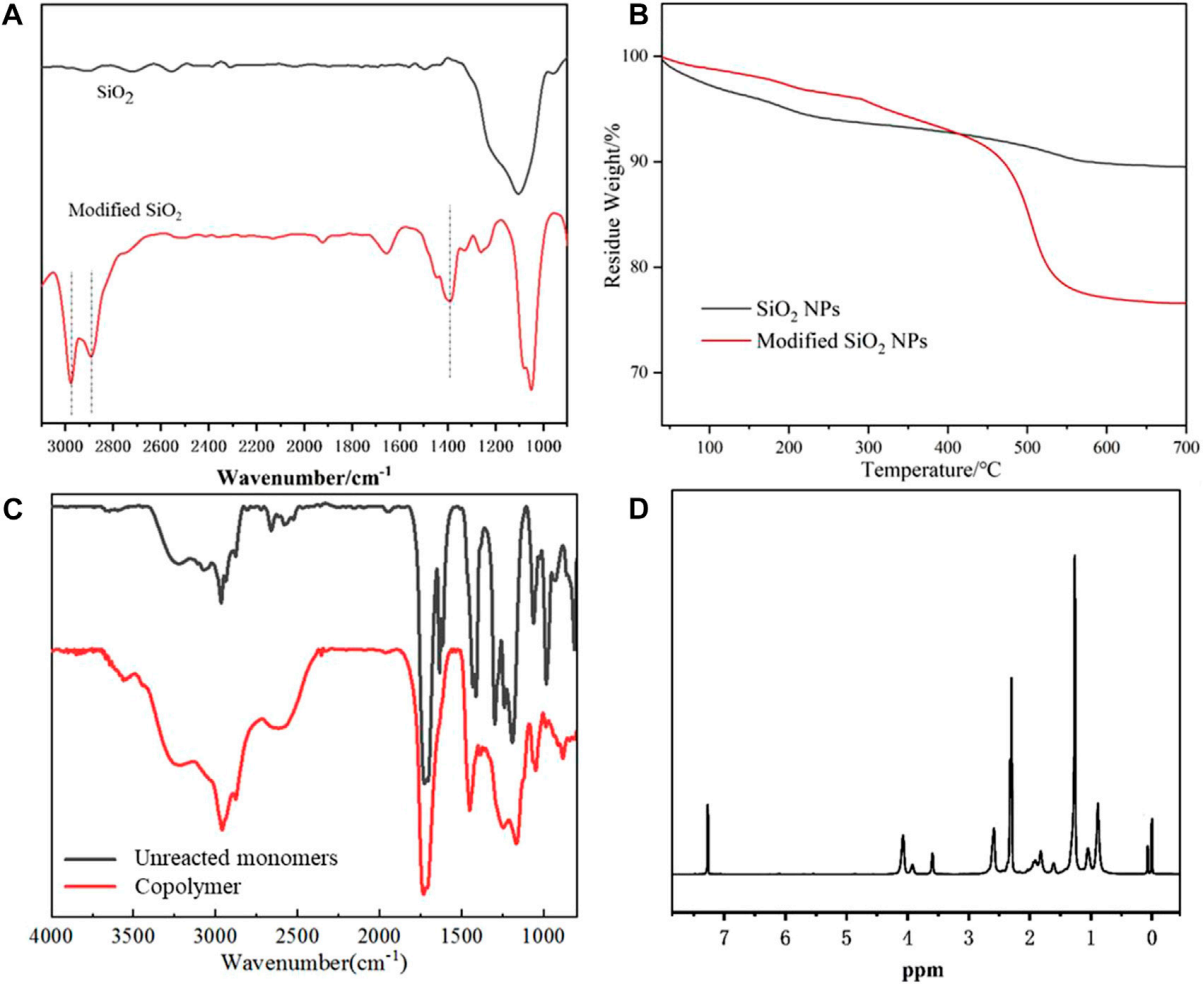
FTIR spectra of SiO_2_ NPs and modified SiO_2_ NPs **(A)**, TGA curves of SiO_2_ NPs and modified SiO_2_ NPs **(B)**, FTIR spectra of unreacted monomers and acrylate copolymer **(C)**, and ^1^H NMR of the acrylate copolymer **(D)**.

The superhydrophobic composite coating was prepared by dispersing GO sheets and modified SiO_2_ NPs in an alcohol solution of acrylate. After the coating is cured, the nanoparticles and microsheets could be evenly embedded in the acrylate copolymer, making the coating both superhydrophobic and wear-resistant. The SiO_2_ NPs were first synthesized through a one-pot sol-gel process and then modified with cetyltrimethoxysilan. The FTIR analysis in Figure 1A shows that the modified SiO_2_ NPs exhibit three new characteristic absorption peaks at 2,861, 2,794 cm^−1^, and 1,463 cm^−1^. The first two peaks correspond to the stretching vibrations of -CH_3_ and -CH_2_-, while the last one is the bending absorption of -CH_2_-, indicating the modification of SiO_2_ NPs. TGA curves in Figure 1B depict the weight loss of unmodified SiO_2_ NPs from 25 to 750°C is only about 8%, while that of modified SiO_2_ exceeds 20%, which also confirms the modification. The size distribution in Supplementary Figure S1 shows the average particle size of as-prepared SiO_2_ NPs is ∼31.08 nm, and the SEM images of purchased GO sheets are shown in Supplementary Figure S2. The FTIR spectra of monomers and acrylate copolymers are depicted in Figure 1C. Obviously, the two peaks corresponding to C=C stretching vibration absorption at 810 and 1,640 cm^−1^ disappeared after polymerization. Cooperating with the 1H NMR result in Figure 1D, it reveals that the acrylate copolymer has been synthesized successfully, and the structure of the copolymer is depicted in Supplementary Scheme S1.

**FIGURE 2:**
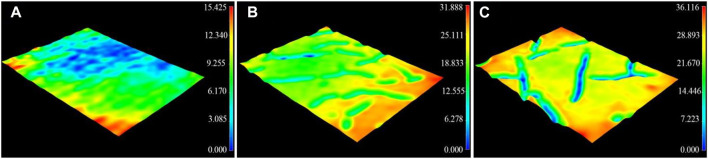
Three-dimensional laser profile images of acrylate copolymer coating with *R*_a_ of 0.175 μm **(A)**, acrylate copolymer/0.25-SiO_2_ NPs coating with *R*_a_ of 5.898 μm **(B)**, and acrylate copolymer/0.25-SiO_2_ NPs/1 wt%-GO sheets coating with *R*_a_ of 6.532 μm **(C)**.

A 3D profilometer was used to investigate the surface morphology of the composite coatings. As shown in Figure 2, roughness of the acrylate copolymer coating, acrylate copolymer/SiO_2_ NPs coating, and acrylate copolymer/SiO_2_–NPs/GO sheet coating are 0.175, 5.898, and 6.532 μm, respectively, which exhibits a significant increase as SiO_2_ NPs and GO sheets embed into copolymer coatings. It suggests that the introduction of nanoparticles and microsheets plays an essential role in building the micro-/nanostructures, thus affecting the superhydrophobicity of the coating.

**FIGURE 3:**
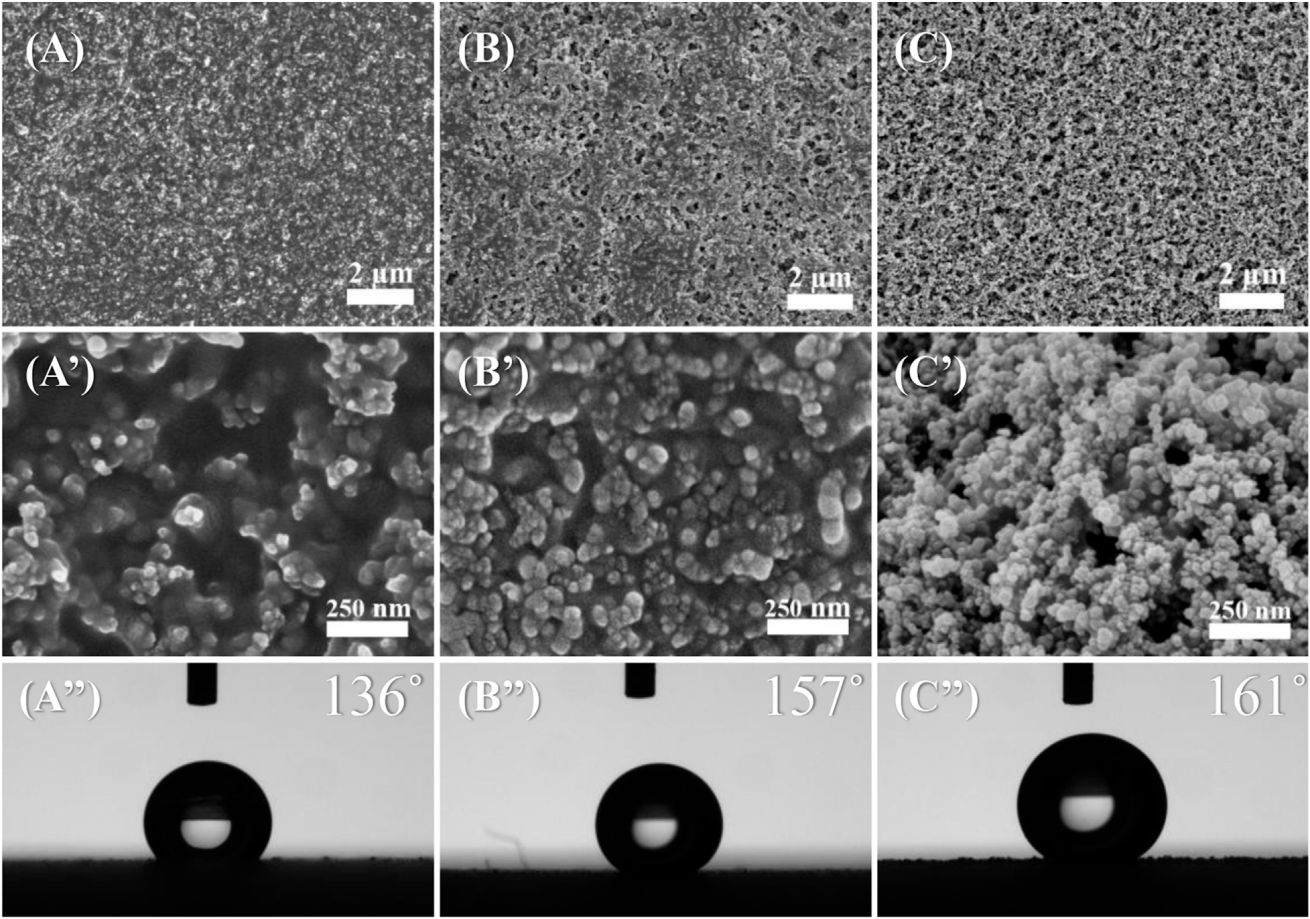
SEM images at different magnifications and WCAs of acrylate copolymer/0.15-SiO_2_ NPs coating **(A,A′,A’’)**, acrylate copolymer/0.25-SiO_2_ NPs coating **(B,B′,B’’)**, and acrylate copolymer/0.35-SiO_2_ NPs coating **(C,C′,C’’)**.

To investigate the possibility of tailoring the surface wettability through composition regulation of the coatings, the surface morphologies and WCA of acrylate copolymer films with different SiO_2_ NP contents were determined. As the amount of added SiO_2_ NPs increased from 0.15 to 0.35 g, more bumps of SiO_2_ NPs appeared on the surface, resulting in an increase in the surface roughness and WCA (Figures 3A–C). The film coating that contains 0.15 g of SiO_2_ NPs failed to achieve superhydrophobicity, which was attributed to the insufficient surface roughness (Figure 3A″). As the amount of added SiO_2_ NPs over 0.25 g, there were many mountain-like protrusions composed of SiO_2_ NP aggregates formed on the surface of the coating, which contribute to the superhydrophobicity with WCA reaching 157° and 161° for the samples with 0.25 and 0.35 g for SiO_2_ NP contents.

**FIGURE 4:**
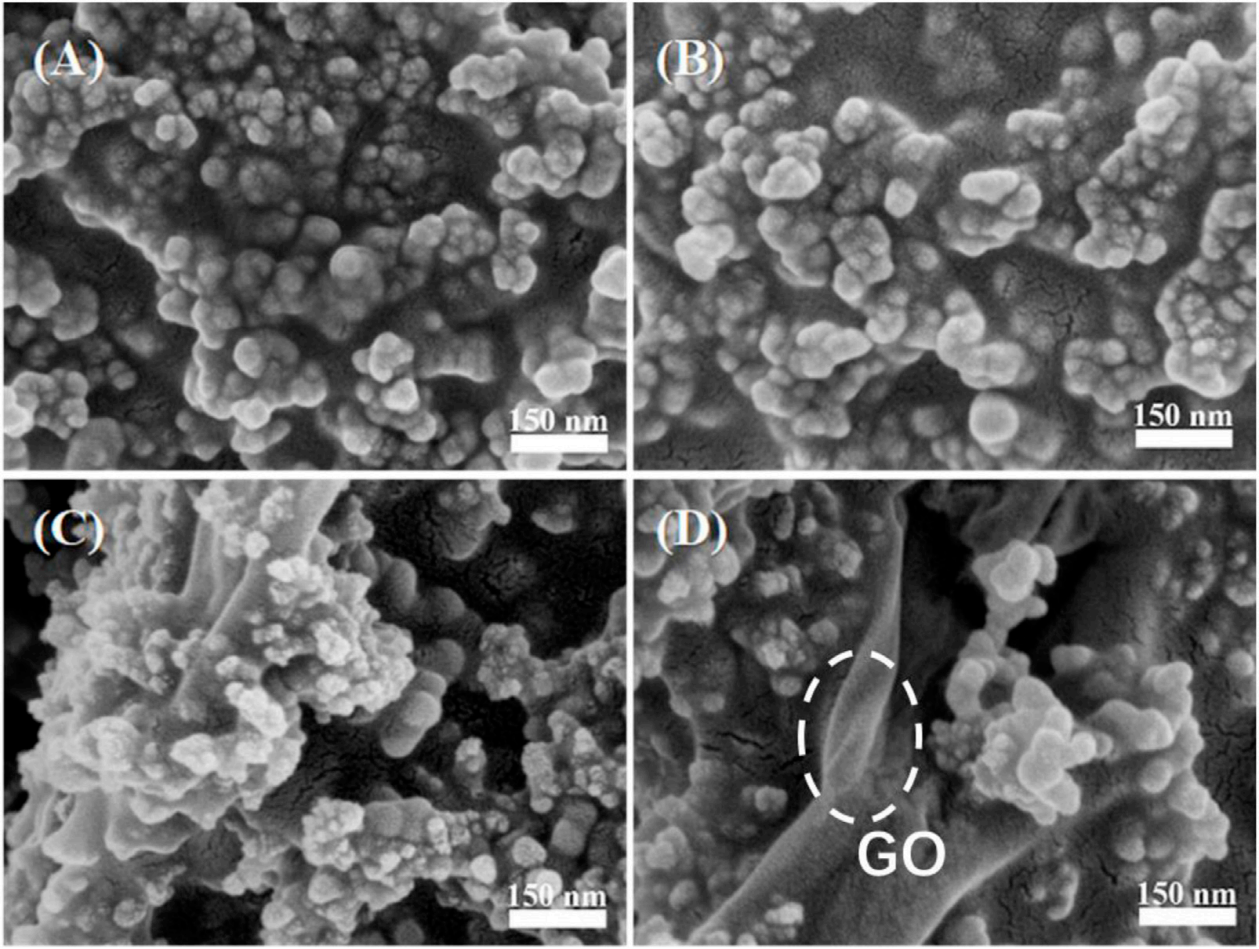
SEM images of composite coatings with 0.25 g SiO_2_ NPs and different GO contents: 0.5 wt% **(A)**, 1% **(B)**, 1.5 wt% **(C),** and 2 wt% **(D).**

To clarify the relationship between GO content and hydrophobicity of the coatings, WCAs of the films with 0.25 g SiO_2_ NPs and different GO contents were measured and listed in Table 1. Interestingly, although the embedded GO sheets may continue to increase the roughness (Figure 2), they did not increase WCA. This is due to the oxygen-containing functional groups on the surface of GO sheets, which weaken the hydrophobicity of the composite film. As shown in Figure 4, with increasing the GO content, more GO sheet surfaces without Si NPs coverage are exposed. Supplementary Figure S3 indicates that the acrylate copolymer/0.25-SiO_2_ NPs/1 wt%-GO sheet composite film could maintain hydrophobicity to several liquids.

**TABLE 1:** WCAs of composite coatings with 0.25 g SiO_2_ NPs and different GO contents.

Sample	Contact angle (˚)					Average value (˚)	Standard deviation
	1	2	3	4	5		
Copolymer	80.4	92.1	86.5	85.2	95.4	87.92	5.9
Copolymer/0.25-SiO_2_	158.2	161.6	149.7	155.1	158.4	158.8	4.5
Copolymer/0.25-SiO_2_/0.5 wt%-GO	155.6	157.5	151.9	158.2	154.4	155.52	2.5
Copolymer/0.25-SiO_2_/1 wt%-GO	159.9	158.2	155.4	157.8	154.9	157.24	2.1
Copolymer/0.25-SiO_2_/1.5 wt%-GO	142.3	141.9	137.5	138.8	131	138.3	4.6
Copolymer/0.25-SiO_2_/2 wt%-GO	120.5	115.2	126.3	119.4	127.9	121.86	5.2

**FIGURE 5:**
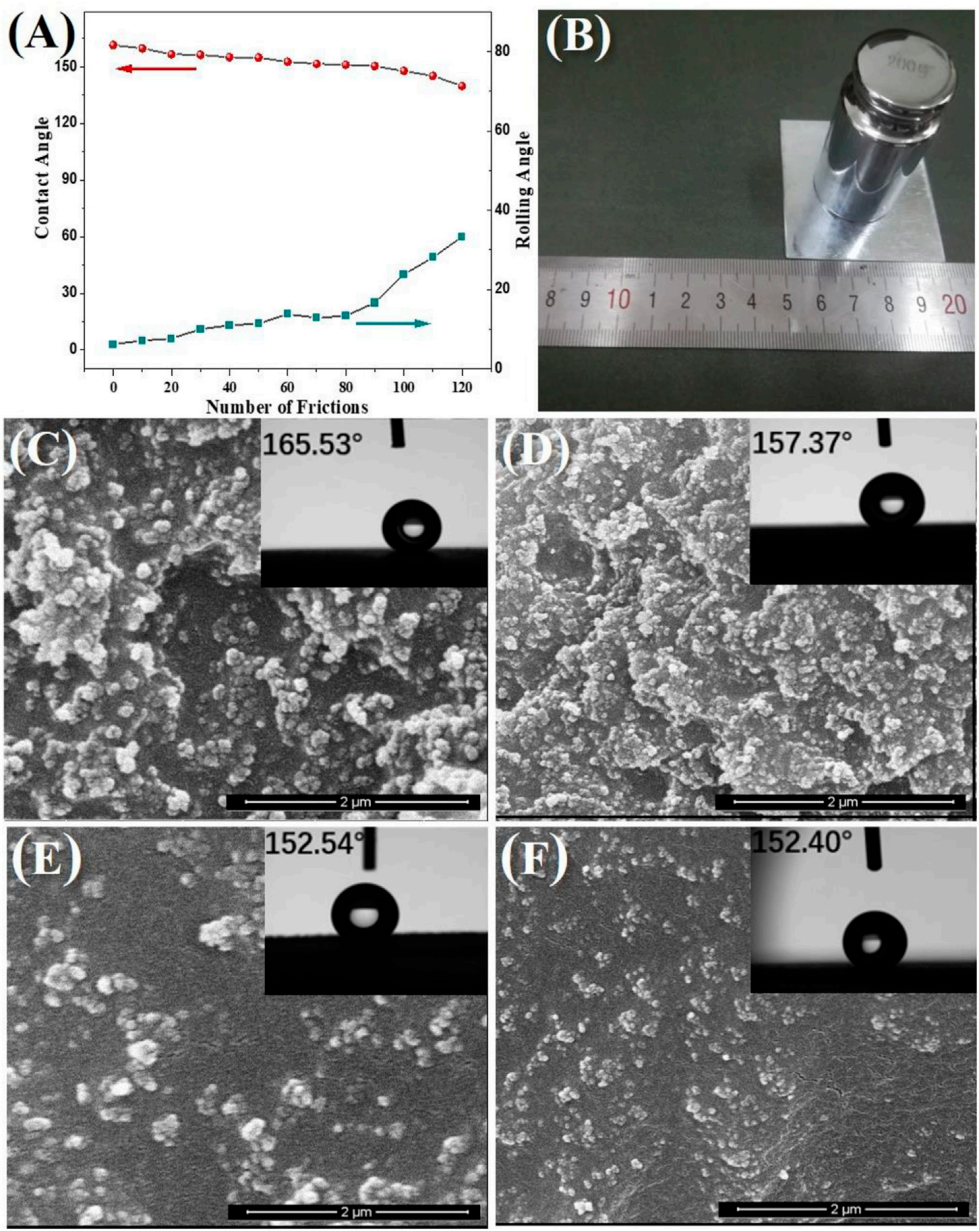
WCAs and WSAs of the acrylate copolymer/SiO_2_–NPs/GO sheets composite coating after different abrasion cycles **(A)**, schematic illustration of the methodology of abrasion test **(B)**, SEM images of the superhydrophobic coating after scraping 0 **(C)**, 30 **(D)**, 60 **(E)**, and 90 **(F)** times.

The long-term durability of the acrylate copolymer/SiO_2_–NPs/GO sheet coating was also evaluated using the sandpaper friction method. During the test, the sandpaper was moved repeatedly from one side to another parallel to the coating surface, and a moving cycle from back to forth was calculated as one abrasion. The sandpaper is 800 mesh and with a load of 200 g, corresponding to a stress of 3.3 kPa. The measured WCAs and WSAs of the composite coating after different abrasion cycles are shown in Figure 5A. It suggests that the composite coating possesses remarkable wear-resistant performance and retains good superhydrophobicity with WCA over 150° after 100 abrasion cycles. SEM images in Figures 5C–F disclose that the superhydrophobic durability of the coating should be ascribed to the uniformity of the composite coating. There are enough new Si NPs and GO sheets exposed on the coated surface to form new micro-/nanostructures after the old ones are scraped off. The XPS results (Supplementary Figure S4) also verify the chemical stability of the coating. These results indicate that the acrylate copolymer/SiO_2_–NPs/GO sheet coating is especially suitable for waterproof and anticorrosion applications under harsh conditions. The cross-hatch test result of the acrylate copolymer/SiO_2_–NPs/GO sheet composite coating is shown in Supplementary Figure S5. Supplementary Table S1 is the comparison of the acrylate copolymer/SiO_2_–NPs/GO sheet composite coating with reported anticorrosion coatings, which demonstrates obvious superiorities of our non-fluorinated superhydrophobic composite coating in high durability and environmental protection.

**FIGURE 6:**
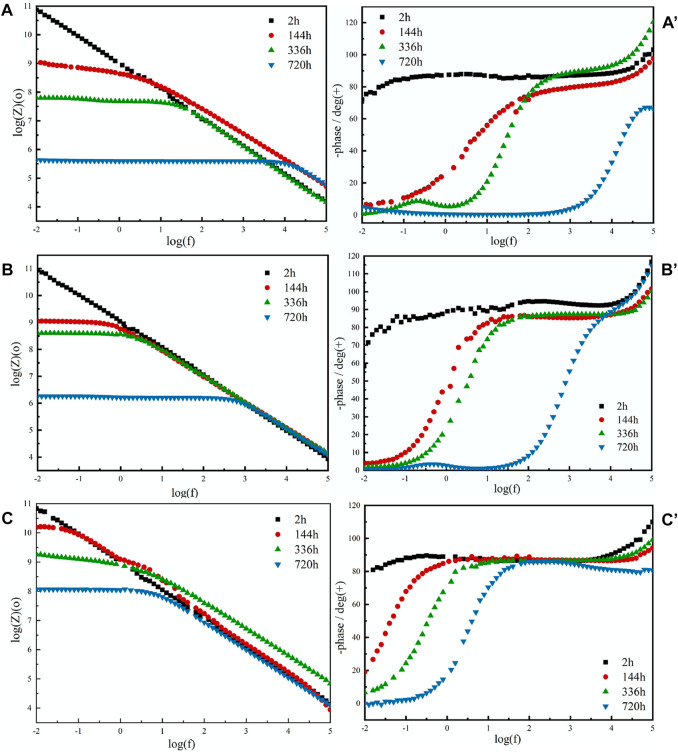
EIS curves of pure acrylate copolymer film coating **(A,A′)**, acrylate copolymer/0.25-SiO_2_ NPs film coating **(B,B′)**, and acrylate copolymer/0.25-SiO_2_ NPs/1 wt%-GO sheets film coating **(C,C′)**.

Electrochemical tests were performed to measure the corrosion resistance of the coating (Wan et al., 2021; Zeng et al., 2022). According to the Bode (log |Z| vs log frequency) curves (Figures 6A–C), the film coating could maintain resistance over a wide frequency range, while the capacitive behavior only occurs at very high frequencies. The impedance values of all film coatings decreased with the increasing immersion time, which is the result of the corrosive electrolyte gradually diffusing into the coating and increasing the porosity and electrolyte pathways. After immersion for 720 h, the |Z|0.01 Hz values of the acrylate copolymer film coating, acrylate copolymer/SiO_2_ NPs film coating, and acrylate copolymer/SiO_2_–NPs/GO sheet film coating decreased to 4.27×10^5^, 1.51×10^6^, and 1.23 × 10^8^ Ω cm^2^, respectively. The |Z|0.01 Hz value of the acrylate copolymer/SiO_2_–NPs/GO sheet film coating is approximately 1,000 times larger than that of the pure acrylate copolymer film coating, revealing the inclusion of SiO_2_ NPs and GO sheets into the acrylate copolymer coating has a significant impact on the corrosion protection performance. Meanwhile, the polarization curves are depicted in Supplementary Figure S6, and the corrosion potential and current density data are listed in Supplementary Table S2.

## Conclusion

In conclusion, a non-fluorinated superhydrophobic coating was successfully fabricated using a facile and environment-friendly method. Electrochemical tests show that the acrylate copolymer/SiO_2_–NPs/GO sheet composite coating exhibits remarkable corrosion resistance, and its |Z|0.01 Hz is approximately 1,000 times that of pure acrylate copolymer coating in a 3.5 wt% NaCl solution. Also, benefiting from the micro-/nanostructures constructed by SiO_2_ NPs and GO sheets on the coating surface, the as-prepared film coating exhibits excellent wear resistance and maintains good superhydrophobicity after 100 abrasion cycles. This method may open a new avenue for the design and fabrication of fluorine-free superhydrophobic surfaces with enhanced anticorrosion and wear-resistant performance.

## Data Availability

The original contributions presented in the study are included in the article/Supplementary Material; further inquiries can be directed to the corresponding author.

## References

[B1] Bayer I. S. (2020). Superhydrophobic coatings from ecofriendly materials and processes: a review. Adv. Mater. Interfaces 7 (13), 2000095. 10.1002/admi.202000095

[B2] Dalawai S. P. Aly M. A. S. Latthe S. S. Xing R. Sutar R. S. Nagappan S. (2020). Recent advances in durability of superhydrophobic self-cleaning technology: a critical review. Prog. Org. Coat. 138, 105381. 10.1016/j.porgcoat.2019.105381

[B3] Ferrari F. Orlando A. Ricci Z. Ronco C. (2019). Persistent pollutants: focus on perfluorinated compounds and kidney. Curr. Opin. Crit. care 25 (6), 539–549. 10.1097/mcc.0000000000000658 31524719

[B4] Ferrari F. Manera M. Mongodi S. Esposito P. Ronco C. (2021). The role of perfluorinated compound pollution in the development of acute and chronic kidney disease. Contrib. Nephrol. 199, 285–296. 10.1159/000517711 34348256

[B5] Fihri A. Bovero E. Al-Shahrani A. Al-Ghamdi A. Alabedi G. (2017). Recent progress in superhydrophobic coatings used for steel protection: A review. Colloids Surfaces A Physicochem. Eng. Aspects 520, 378–390. 10.1016/j.colsurfa.2016.12.057

[B6] Ghasemlou M. Daver F. Ivanova E. P. Adhikari B. (2019). Bio-inspired sustainable and durable superhydrophobic materials: from nature to market. J. Mater. Chem. A Mater. 7 (28), 16643–16670. 10.1039/c9ta05185f

[B7] Hooda A. Goyat M. Pandey J. K. Kumar A. Gupta R. (2020). A review on fundamentals, constraints and fabrication techniques of superhydrophobic coatings. Prog. Org. Coat. 142, 105557. 10.1016/j.porgcoat.2020.105557

[B8] Kang L. Shi L. Zeng Q. Liao B. Wang B. Guo X. J. S. (2021). Melamine resin-coated lignocellulose fibers with robust superhydrophobicity for highly effective oil/water separation. Sep. Purif. Technol. 279, 119737. 10.1016/j.seppur.2021.119737

[B9] Latthe S. S. Sutar R. S. Kodag V. S. Bhosale A. Kumar A. M. Sadasivuni K. K. (2019a). Self–cleaning superhydrophobic coatings: Potential industrial applications. Prog. Org. Coat. 128, 52–58. 10.1016/j.porgcoat.2018.12.008

[B10] Latthe S. S. Sutar R. S. Bhosale A. K. Nagappan S. Ha C.-S. Sadasivuni K. K. (2019b). Recent developments in air-trapped superhydrophobic and liquid-infused slippery surfaces for anti-icing application. Prog. Org. Coat. 137, 105373. 10.1016/j.porgcoat.2019.105373

[B11] Mao Y. Huang Q. Meng B. Zhou K. Liu G. Gugliuzza A. (2020). Roughness-enhanced hydrophobic graphene oxide membrane for water desalination via membrane distillation. J. Membr. Sci. 611, 118364. 10.1016/j.memsci.2020.118364

[B12] Naderizadeh S. Athanassiou A. Bayer I. S. (2018). Interfacing superhydrophobic silica nanoparticle films with graphene and thermoplastic polyurethane for wear/abrasion resistance. J. Colloid interface Sci. 519, 285–295. 10.1016/j.jcis.2018.02.065 29505990

[B13] Nguyen-Tri P. Tran H. N. Plamondon C. O. Tuduri L. Vo D.-V. N. Nanda S. (2019). Recent progress in the preparation, properties and applications of superhydrophobic nano-based coatings and surfaces: A review. Prog. Org. Coat. 132, 235–256. 10.1016/j.porgcoat.2019.03.042

[B14] Shi Z. Ouyang Y. Qiu R. Hu S. Zhang Y. Chen M. (2019). Bioinspired superhydrophobic and oil-infused nanostructured surface for Cu corrosion inhibition: a comparison study. Prog. Org. Coat. 131, 49–59. 10.1016/j.porgcoat.2019.02.004

[B15] Sun K. Yang H. Xue W. He A. Zhu D. Liu W. (2018). Anti-biofouling superhydrophobic surface fabricated by picosecond laser texturing of stainless steel. Appl. Surf. Sci. 436, 263–267. 10.1016/j.apsusc.2017.12.012

[B16] Wan S. Chen H. Ma X. Chen L. Lei K. Liao B. (2021). Anticorrosive reinforcement of waterborne epoxy coating on Q235 steel using NZ/BNNS nanocomposites. Prog. Org. Coat. 159, 106410. 10.1016/j.porgcoat.2021.106410

[B17] Wang S. Urban M. W. (2020). Self-healing polymers. Nat. Rev. Mater. 5 (8), 562–583. 10.1038/s41578-020-0202-4

[B18] Wang X. Pan Y. Liu X. Liu H. Li N. Liu C. (2019). Facile fabrication of superhydrophobic and eco-friendly poly (lactic acid) foam for oil–water separation via skin peeling. ACS Appl. Mater. Interfaces 11 (15), 14362–14367. 10.1021/acsami.9b02285 30916921

[B19] Wei D. Wang J. Liu Y. Wang D. Li S. Wang H. (2021). Controllable superhydrophobic surfaces with tunable adhesion on Mg alloys by a simple etching method and its corrosion inhibition performance. Chem. Eng. J. 404, 126444. 10.1016/j.cej.2020.126444

[B20] Xu M. Grabowski A. Yu N. Kerezyte G. Lee J.-W. Pfeifer B. R. (2020). Superhydrophobic drag reduction for turbulent flows in open water. Phys. Rev. Appl. 13 (3), 034056. 10.1103/physrevapplied.13.034056

[B21] Zeng Y. Kang L. Wu Y. Wan S. Liao B. Li N. (2022). Melamine modified carbon dots as high effective corrosion inhibitor for Q235 carbon steel in neutral 3.5 wt% NaCl solution. J. Mol. Liq. 349, 118108. 10.1016/j.molliq.2021.118108

