# Supplementary material for: A non-fluorinated superhydrophobic composite coating with excellent anticorrosion and wear-resistant performance
Source: Front Chem. 2022 Oct 3;10:952919. doi: 10.3389/fchem.2022.952919 (PMC9574401; doi:10.3389/fchem.2022.952919)
Supplement: Supplementary file 1 [file DataSheet1.docx]

**Supporting information**

**A non-fluorinated superhydrophobic composite coating with excellent** **anti-corrosion and wear-resistant performance**

*Peng Xiao*, Liheng Yang, Jianjun Liu, Xiaoqin Zhang, Dabing Chen*

*State Grid Jiangsu Electric Power Co., Ltd. Research Institute, Nanjing, 211103, Jiangsu, P. R. China*

*Corresponding Author: Dr. Peng Xiao, Email: vodoco@foxmail.com.


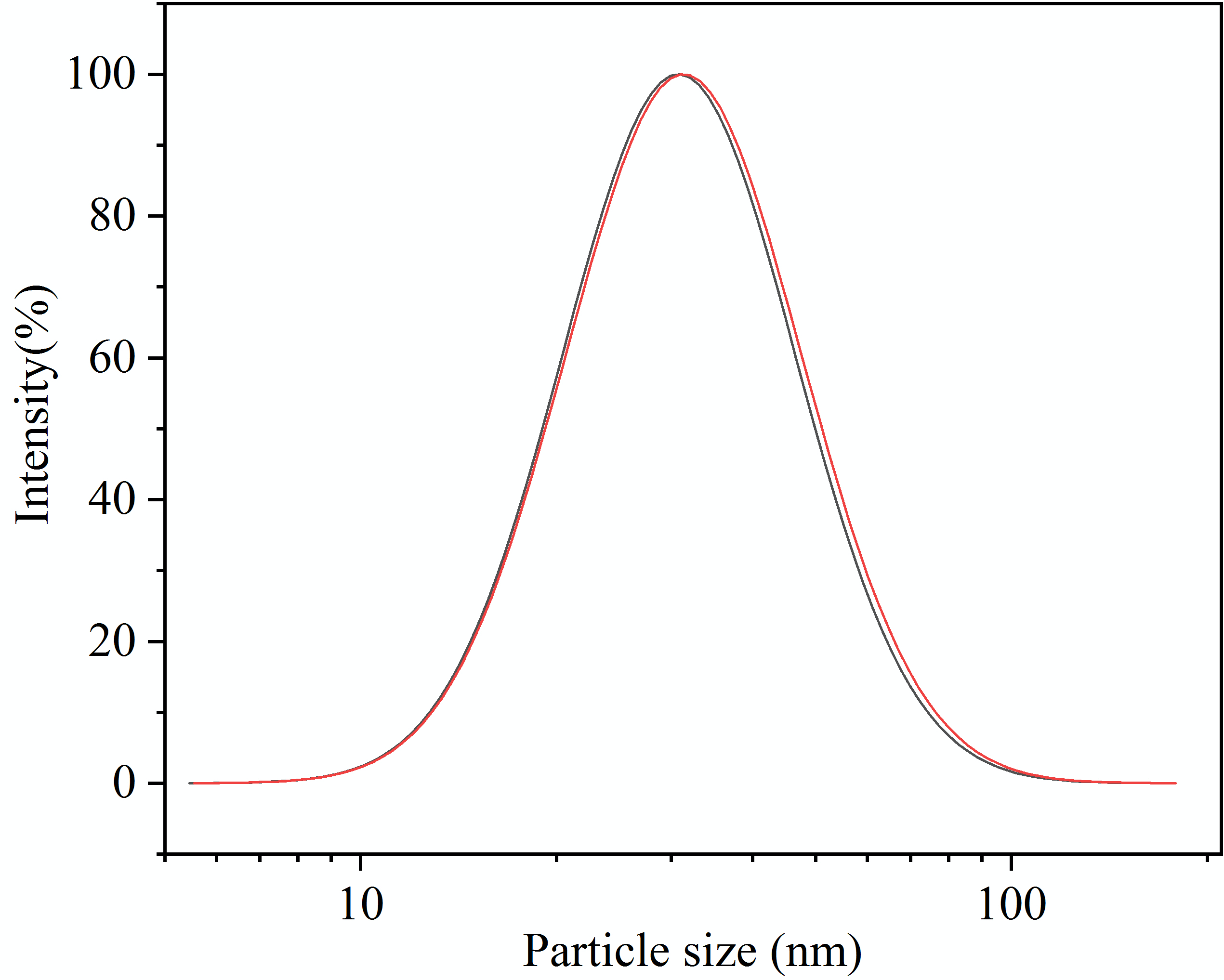


**FIGURE S1** Curves of size-distribution of SiO_2_ NPs and the average particle size of SiO_2_ NPs is 31.08 nm (std. err. 2.68).


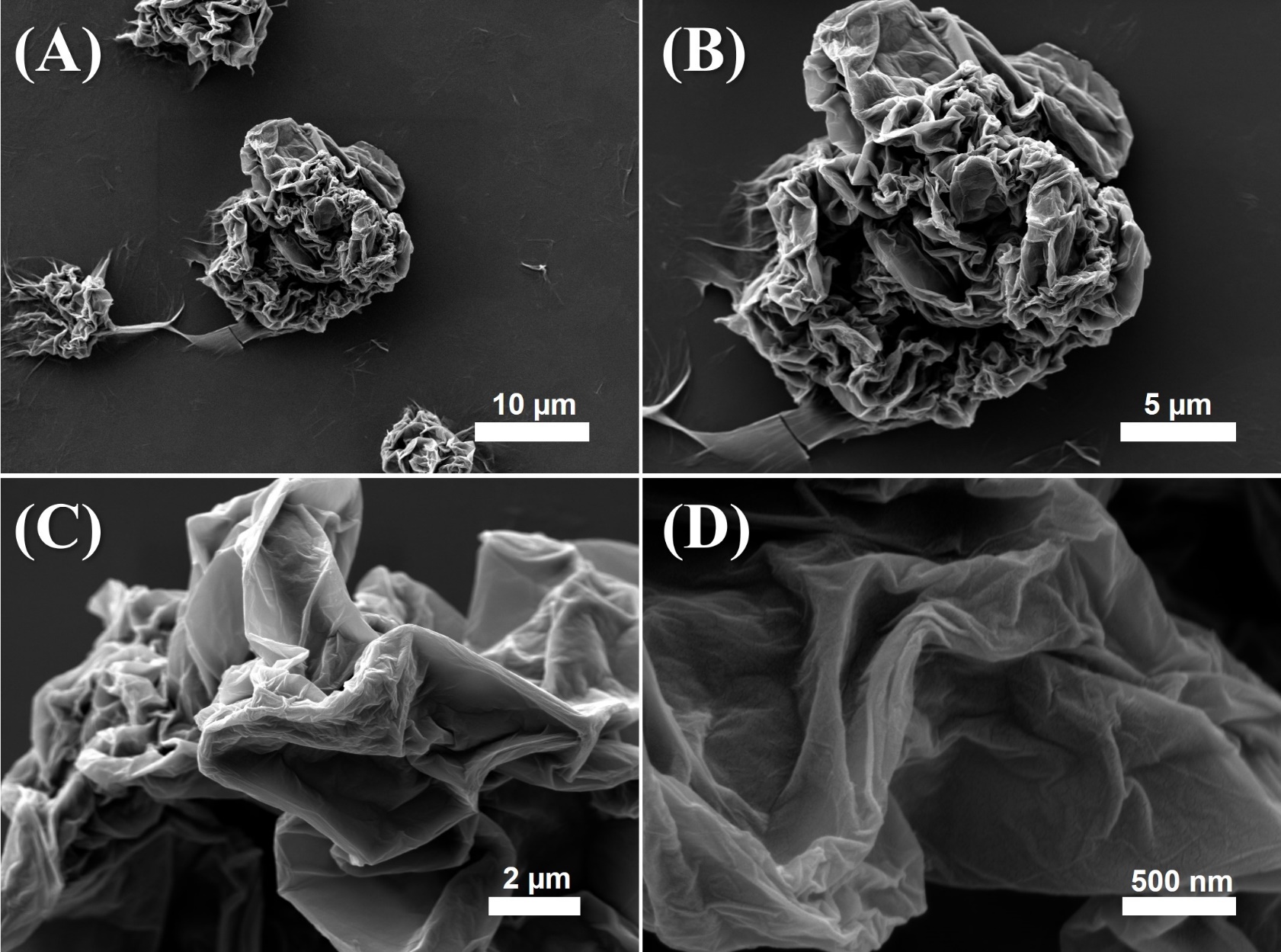


**FIGURE S2** SEM images of purchased GO: (A)meg. 5000 (B)meg. 10,000. (C)meg. 20,000. and (D)meg. 100,000.


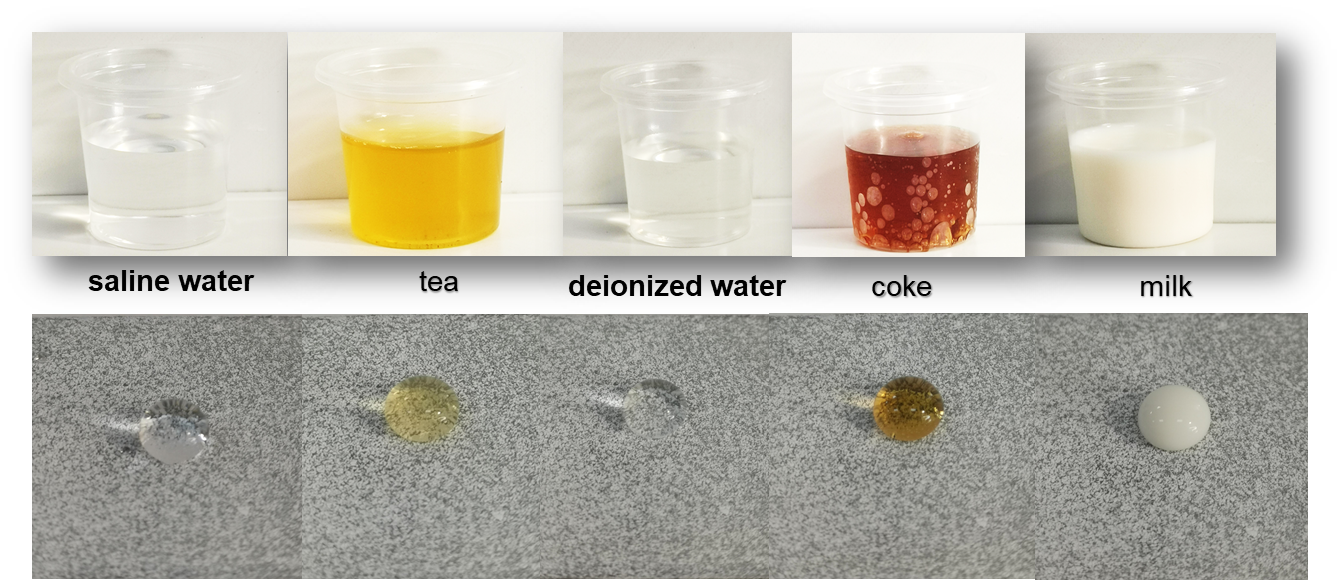


**FIGURE S3** Images of 3.5% NaCl solution, tea, water, coke and milk on acrylate copolymer/SiO_2_ NPs/GO sheets coating film.


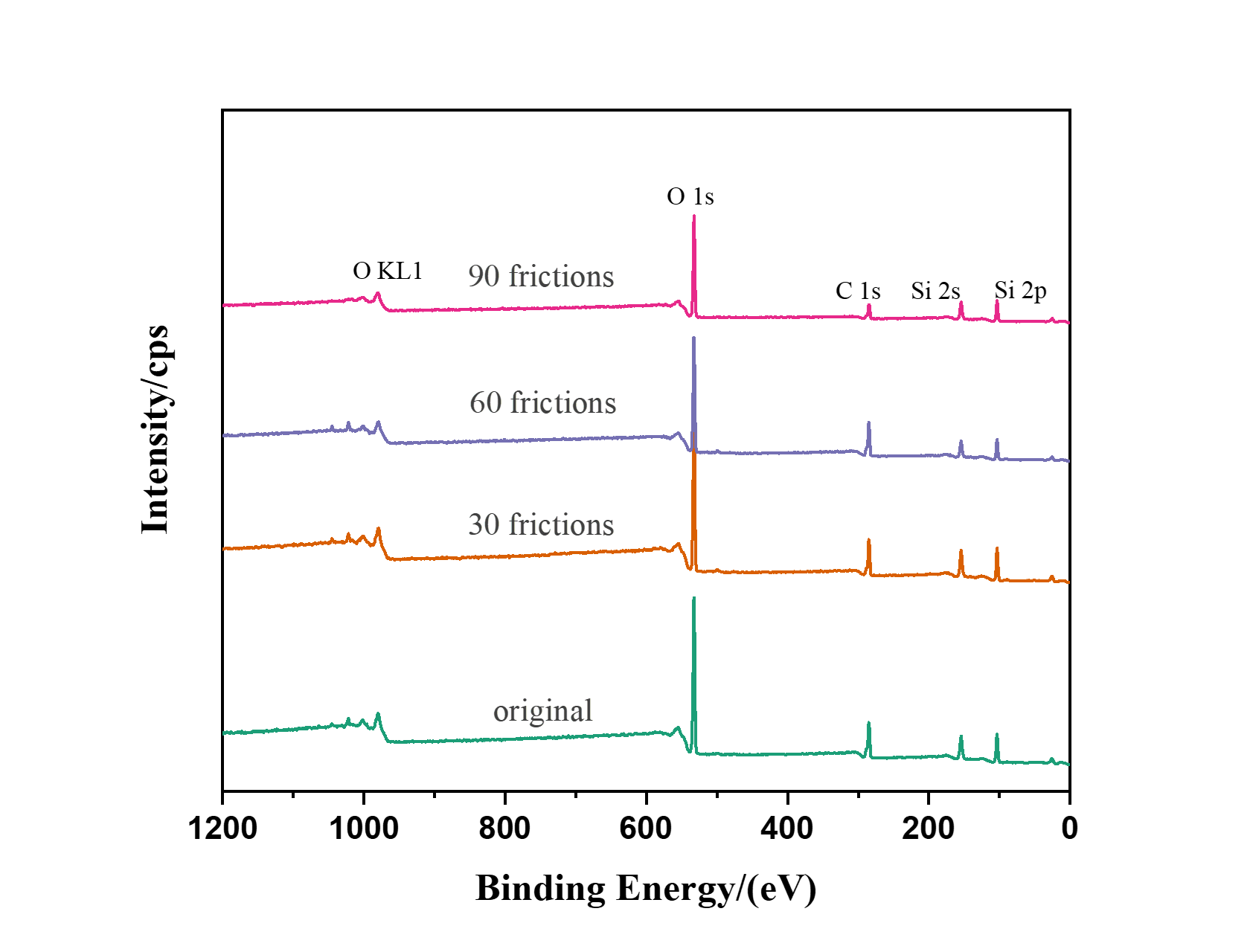


**FIGURE S4** XPS survey spectra of the prepared the acrylate copolymer/SiO_2_ NPs/GO sheets composite coating after scratched by sandpaper 0, 30, 60 and 90 times, respectively.


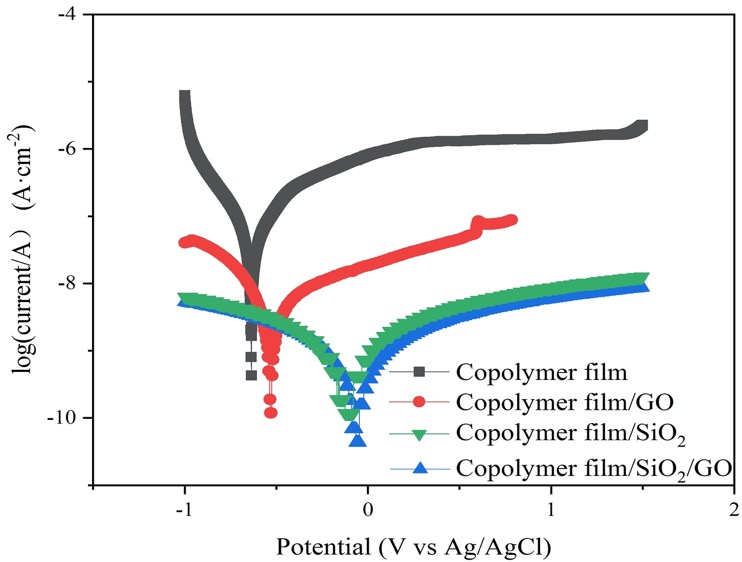


**FIGURE S5** Polarization curves of copolymer coating, copolymer/SiO_2_ NPs coating, and copolymer/SiO_2_ NPs/GO sheets coating after 720h of immersion in 3.5 wt.% NaCl solution.


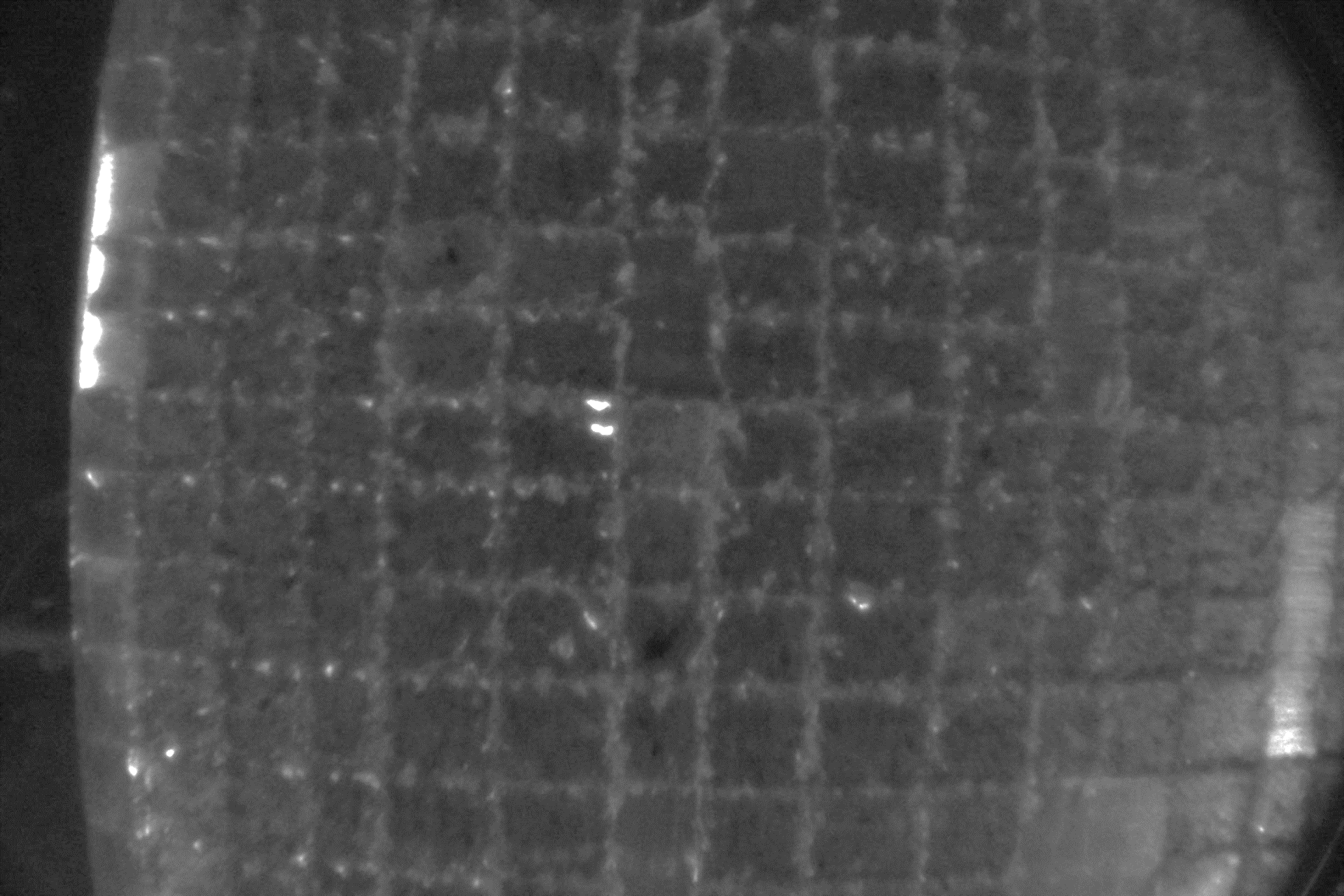


**FIGURE S6**. Cross-hatch test result of the as-prepared copolymer/0.25-SiO_2_/1 wt%-GO coating.

**SCHEME S1**. The structure of acrylate copolymer

**Table S1**. Corrosion potential (*E*_corr_) and Corrosion current density (*I*_corr_) of different samples after 288h of immersion in 3.5 wt.% NaCl aqueous solution.

| Sample | *E*_corr_ (mV vs. SCE) | *I*_corr_ (A/cm^2^) |
| --- | --- | --- |
| copolymer coating | -655 | 3.16×10^-7^ |
| copolymer/GO sheets coating | -186 | 7.41×10^-9^ |
| copolymer/SiO_2_ NPs coating | -92 | 1.01×10^-9^ |
| copolymer/SiO_2_ NPs/GO sheets coating | -54 | 5.89×10^-10^ |

**Table S2 Comparison table of some related works in last 10 years.**

| ecofriendly/method | abrasion length | corrosion output substrate | year | references |
| --- | --- | --- | --- | --- |
| yes/spraying | 20 m | tinplate | 2022 | our work |
| yes/electrochemical | -. | galvanized steel | 2021 | [1] |
| yes/deposition | -. | aluminum substrate | 2020 | [2] |
| yes/brushing | 20 m | concrete block substrate | 2019 | [3] |
| yes/electroplating | 0.1 m | steel | 2018 | [4] |
| fluoro related was used | 6 m | copper substrate | 2017 | [5] |
| yes/spraying | -. | 1045 steel | 2016 | [6] |
| HF was used | 1 m | S45C steel substrate | 2015 | [7] |
| yes/spraying | - | stainless steel | 2014 | [8] |
| yes/electrodeposition | 0.07 m | magnesium alloy | 2013 | [9] |
| yes/electrodeposition | -. | stainless steel | 2012 | [10] |

**References**

[1] Polyakov, N. A., et al. "Formation and anticorrosion properties of superhydrophobic zinc coatings on steel." Chemical Engineering Journal 421 (2021): 127775.

[2] Xu, Shuangshuang, Qing Wang, and Ning Wang. "Eco-friendly fabrication of superhydrophobic surface with anti-corrosion by transferring dendrite-like structures to aluminum substrate." Colloids and Surfaces A: Physicochemical and Engineering Aspects 595 (2020): 124719.

[3] Song, Jinlong, et al. "Inexpensive and non-fluorinated superhydrophobic concrete coating for anti-icing and anti-corrosion." Journal of colloid and interface science 541 (2019): 86-92.

[4] XIANG, Tengfei, et al. Fabrication of inherent anticorrosion superhydrophobic surfaces on metals. ACS Sustainable Chemistry & Engineering, 2018, 6.4: 5598-5606.

[5] Yang, Zhengqing, et al. "Superhydrophobic epoxy coating modified by fluorographene used for anti-corrosion and self-cleaning." Applied Surface Science 401 (2017): 146-155.

[6] Pan, Sai, et al. "Fabrication of superhydrophobic coating via spraying method and its applications in anti-icing and anti-corrosion." Applied Surface Science 389 (2016): 547-553.

[7] Zhang, Hongmei, et al. "Fabrication of superhydrophobic textured steel surface for anti-corrosion and tribological properties." Applied Surface Science 359 (2015): 905-910.

[8] Chen, Xiuyong, et al. "Large-scale fabrication of superhydrophobic polyurethane/nano-Al2O3 coatings by suspension flame spraying for anti-corrosion applications." Applied Surface Science 311 (2014): 864-869.

[9] She, Zuxin, et al. "Researching the fabrication of anticorrosion superhydrophobic surface on magnesium alloy and its mechanical stability and durability." Chemical Engineering Journal 228 (2013): 415-424.

[10] de Leon, Al Christopher C., Roderick B. Pernites, and Rigoberto C. Advincula. "Superhydrophobic colloidally textured polythiophene film as superior anticorrosion coating." ACS applied materials & interfaces 4.6 (2012): 3169-3176.
